# Underwater sound to probe sea ice melting in the Arctic during winter

**DOI:** 10.1038/s41598-020-72917-4

**Published:** 2020-09-29

**Authors:** Madan M. Mahanty, G. Latha, R. Venkatesan, M. Ravichandran, M. A. Atmanand, A. Thirunavukarasu, G. Raguraman

**Affiliations:** 1 National Institute of Ocean Technology, Ministry of Earth Sciences, Chennai, India; 2 National Centre for Polar and Ocean Research, Ministry of Earth Sciences, Goa, India

**Keywords:** Ocean sciences, Physical oceanography

## Abstract

Over a 4-year period between 2015 and 2019, in-situ time series measurements of ocean ambient noise over the frequency range 100 Hz to 10 kHz, by an autonomous passive acoustic monitoring system have been made in the Kongsfjorden, Svalbard, Arctic. We characterize the noise due to sea ice melting during winter (December–January). This unique observation reveals loud noise signatures, of the order of 8 dB higher than the background noise, showing the signature of sea ice melting. Such observations are crucial for monitoring sea ice melting, especially during winter, to understand the recent warming of Arctic waters. The anomalous air temperature due to local atmospheric forcing and warming of ocean temperature in the fjord through ocean tunneling, individually or combinedly, is responsible for such sea ice melting. The cyclonic events in the Arctic are responsible for the anomalous atmospheric and ocean conditions, causing sea ice melting in winter.

## Introduction

Arctic fjords are critical locations that have an impact on the stability of the glacier and are characterized by dynamic ice-ocean boundary^1^. Many researchers have been studied on recent warming of glacier fjords due to the intrusion of warm seawater from the ocean shelf, which leads to submarine melting^2–4^. Previous studies have also focused on warming surface air temperature above freezing, resulting in winter sea ice melting, and are associated with the passage of anomalous extreme cyclone events^5–13^. A recent record of surface air temperature above 7 °C and low sea ice concentration was observed after the extreme North Atlantic windstorm ‘Frank’ entered into the Arctic Circle^13^ during the period 29 December 2015 to 02 January 2016. This rapid rate of glacier melting contributes to the global sea level rise^14^.

The methods that are commonly used in these studies include satellite imaging and short-term oceanographic measurements^15,16^, but they are still limited in terms of temporal and spatial resolution as well as in harsh weather conditions. Ice-ocean dynamic processes are poorly studied, particularly during the winter period due to lack of sunlight during polar nights with extreme cyclonic events. A technique which overcomes these issues, is passive acoustic measurements and monitoring that uses naturally generated sounds to obtain insight about sources of underwater noise^17–23^. Previous studies have hypothesized that air-filled pores in glacier ice are resulting from the bulk compression of snow layers over time^24^, whereas in sea ice, gas exists as air bubbles when seawater becomes supersaturated in relation to the interface of ocean-sea ice-atmosphere^25–28^. It is also reported that as the temperature decreases with increasing thickness of ice cover air bubbles are generated and the entrapped tiny air bubbles get released when sea ice melts^25,26^. The content of air bubbles in the sea ice is smaller than in the glacier ice^29^. It has been experimentally proven by making measurements directly by melting a sample of first year sea ice from the Arctic^30^. The sound produced due to release of tiny air bubbles during sea ice melting, along with excellent sound propagating properties of seawater, become a good mechanism for measuring the sea ice melting using passive acoustic technique. The acoustic sensing of glacier ice melting in the Arctic and subarctic seas in summer have been studied by many researchers^31–36^. However, the anomalous winter sea ice melting noise in the Arctic and the contributing factors have not been studied.

To address these issues, we conducted the time series passive acoustic measurements at Kongsfjorden, an open fjord in the Arctic with both Atlantic and glacial influences, Svalbard^37,38^, for the period of 2015–2019, by deployment and maintenance of the autonomous ambient noise system as part of IndArc mooring system^39^. We have analyzed the interannual variability of ambient noise during the winter. This study reveals that sea ice melting sounds are anomalous in winter and strongly related with positive anomaly of water temperature due to warm seawater entering the fjord as well as the anomalous warming in surface air temperature.

## Results

### Analysis of sea ice melting sound

**Figure 1:**
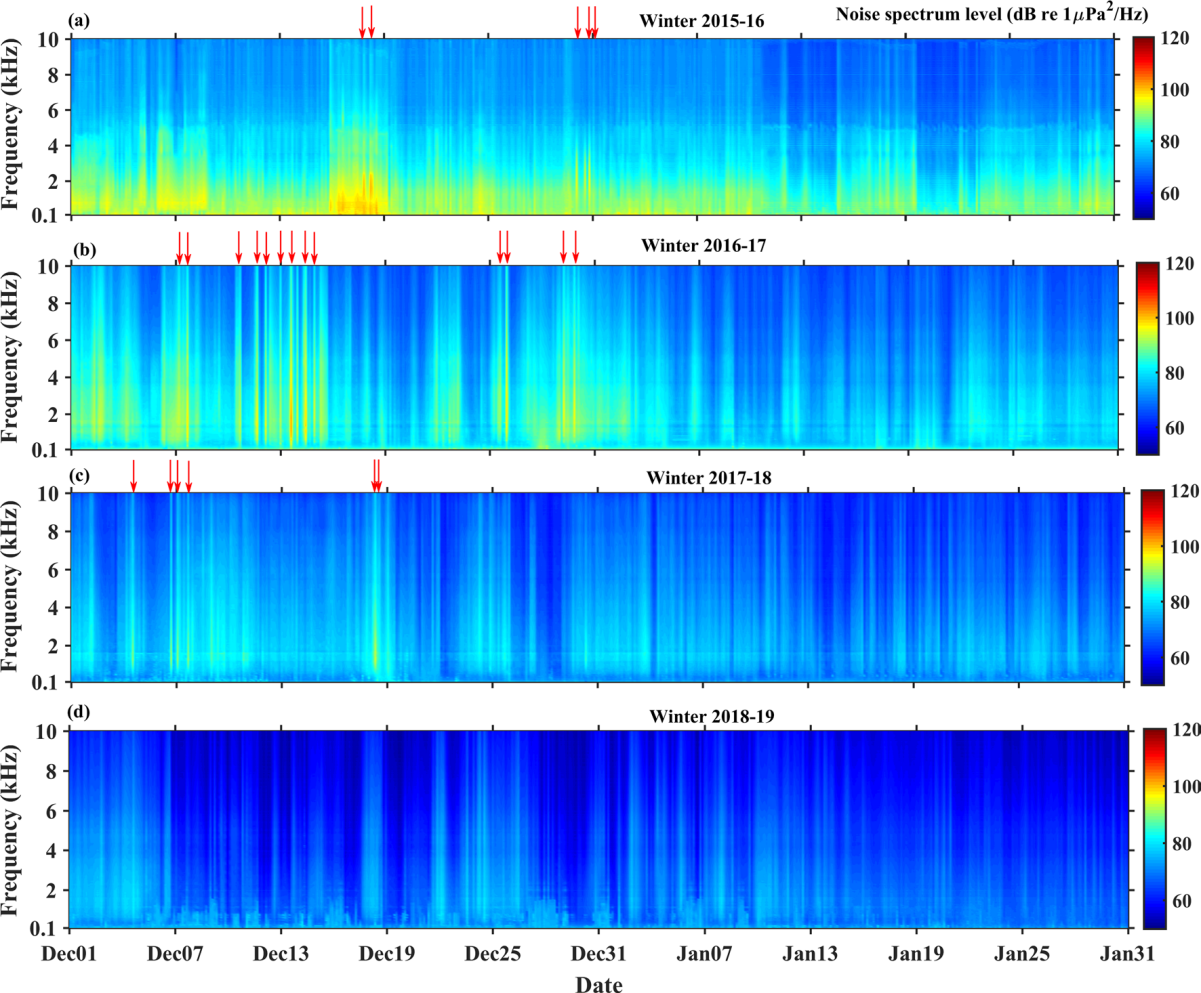
The spectrogram of ambient noise levels in the Kongsfjorden during (**a**) winter 2015–16, (**b**) winter 2016–17, (**c**) winter 2017–18, and (**d**) winter 2018–19. The red arrows indicate sea ice melting events with spectral peak in 1–3 kHz.

In this study, measurements made for 62 days in each winter period were considered for analysis. All the acoustic data were converted into audio files and analyzed using the time–frequency spectrograms to study the sea ice melting sound. The audio files relating to sea ice melting show a loud and impulsive popping noise by breaking of tiny air bubbles entrapped in the ice (Supplementary Audio S1). It is observed that sea ice melting sounds were recorded during the passage of extreme cyclonic events. A total of 24 data sets were recorded during the study period; 5 were recorded in winter 2015–16, 13 were recorded in 2016–17 and 6 data sets recorded in winter 2017–18, and no acoustic signature of ice melting sound in the winter 2018–19. The spectrogram in Fig. 1 shows the time series of interannual variability of ambient noise levels in winter in the Kongsfjorden. It is observed that noise spectra due to sea ice melting is very distinct, and ~ 8 dB higher than the subtle background noise level (Fig. 1). The red arrows in Fig. 1 indicate the number of sea ice melting sounds anomalous in each wintertime, with a spectral peak between 1 and 3 kHz, and a maximum of ~ 98 dB re 1 μPa^2^/Hz. However, the spectral content extends with low intensities in the frequency range up to 10 kHz. The anomalous sea ice melting sounds correspond to high surface air temperature as well as the water temperature in the Kongsfjorden, and each of these are detailed below.

### Sea ice melting sounds associated with surface air temperature

**Figure 2:**
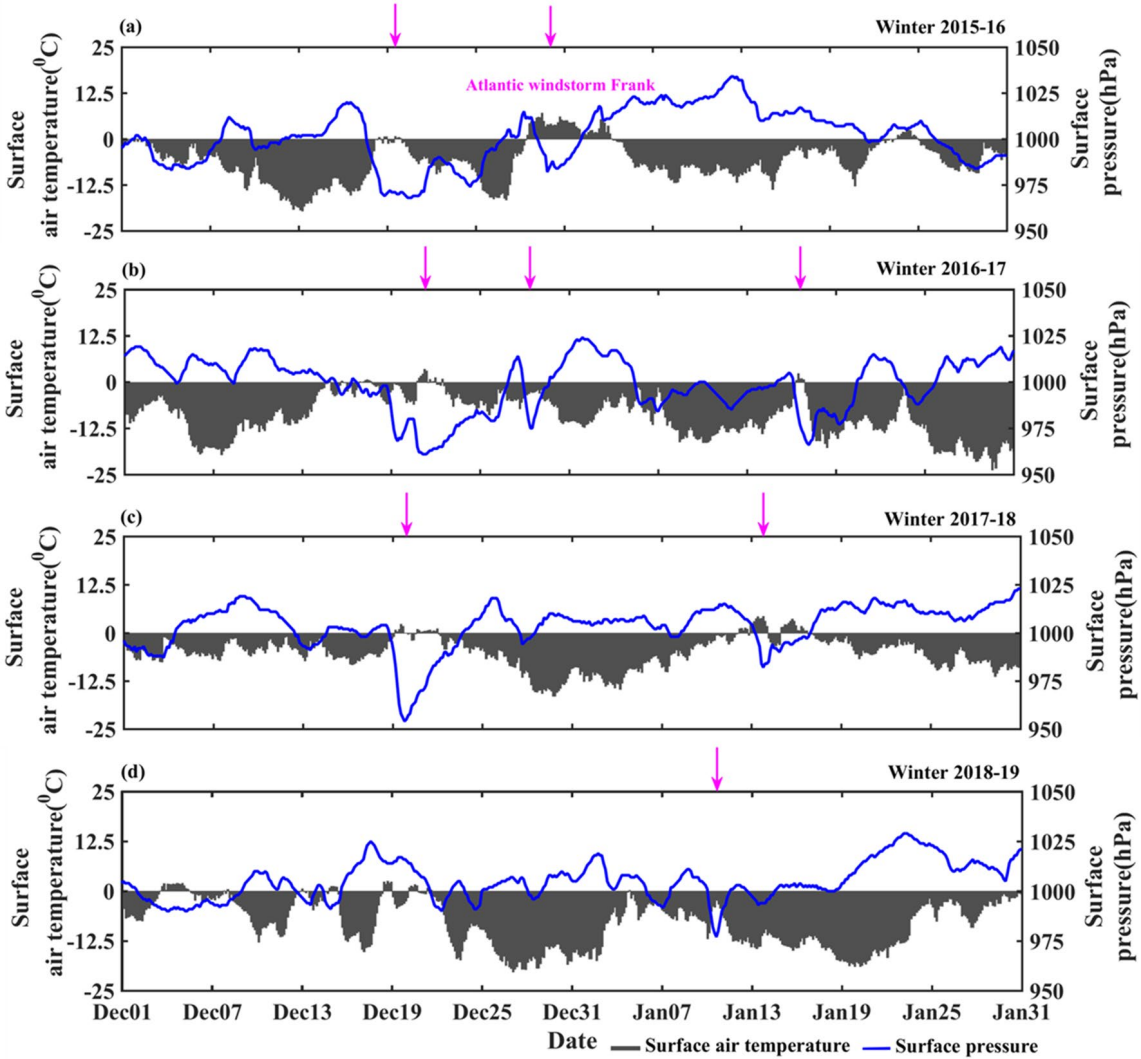
Meteorological observations in winter at Ny-Alesand (**a**) surface air temperature (°C) and surface pressure (hPa) during (**a**) 2015–16, (**b**) 2016–17, (**c**) 2017–18, and (**d**) 2018–19. The arrows indicate passage of the extreme cyclone event.

Figure 2 shows the time series of interannual variability of surface air temperature along with the surface pressure in winter at the Ny-Alesund closer to the IndArc mooring. Low pressure occurrences during extreme cyclone events are indicated (in magenta arrows) each winter period. It is observed that two extreme cyclone events occurred in winter 2015–16, with a minimum surface pressure of 968 hPa on 18th December 2015, and the most record-breaking extreme Atlantic windstorm ‘Frank’ occurred during 29 December 2015 to 03 January 2016. In Fig. 2, it is shown that immediately preceding the Atlantic windstorm, the air temperature was below freezing for several days. It is clearly noticed that air warmed up significantly for the duration of 6 days during the event. The fjord may not have thicker sea ice and hence few degree increase in surface air temperature, resulted in a sea-ice melt. The sea ice melting occurred on 30th December 2015 (though the increase in air temperature started on 29th December 2015) associated with a warming in surface air temperature (7 °C), during the passage of extreme North Atlantic windstorm ‘Frank’ (Fig. 2). In winter 2016–17, three cyclone events occurred with extreme low pressure on 21st December 2016, 28th December 2016 and 15th January 2017 as shown in Fig. 2b.

In 2017–18, two cyclone events were observed on 20th December 2017 and 14th January 2018 (Fig. 2c), that stirred and advected the shelf warm seawater into the Kongsfjorden through surface turbulent process and resulted in sea ice melting. In 2018–19, a single low pressure of 980 hPa was observed on 12th January 2019 (Fig. 2d), and no sea ice melting sounds have been recorded in association with the extreme event.

### Sea ice melting sound associated with warm seawater intrusion

**Figure 3:**
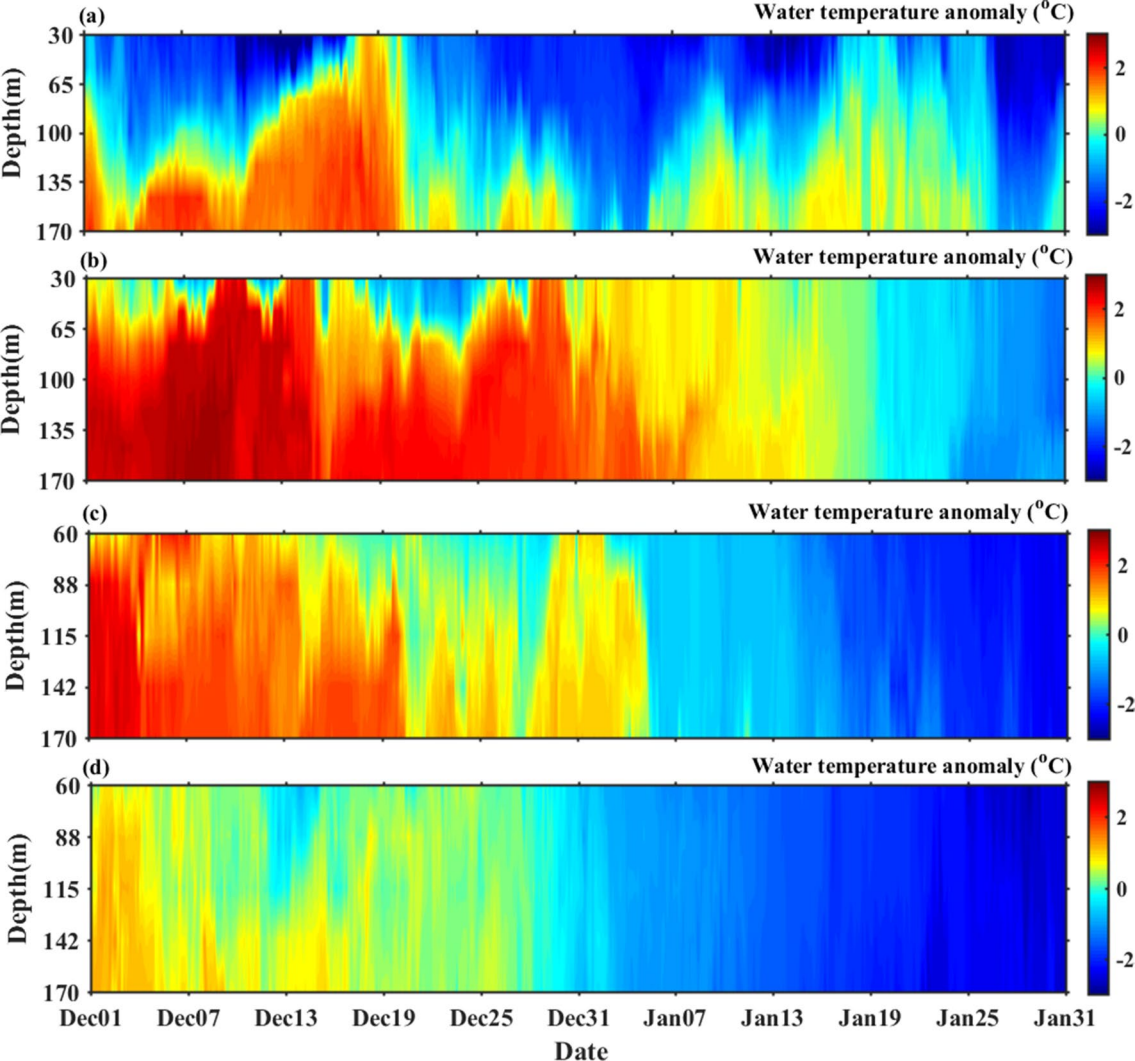
Water temperature anomalies (°C) at IndArc mooring in the Kongsfjorden in winter with a minimum depth of 30 m during (**a**) 2015–16 and (**b**) 2016–17, and 60 m during (**c**) 2017–18 and (**d**) 2018–19.

Figure 3 shows the time series of interannual variability of water temperature anomalies in winter in the Kongsfjorden. The data show a strong variation in water temperature anomalies, and are commonly associated with prior to and during the passage of extreme cyclonic events. In Fig. 3a, the water temperature during 17–19 December 2015, shows a period of warming in the Kongsfjorden. On 18th December 2015, an extreme cyclone event crossed the IndArc mooring location and show a strong positive temperature anomaly due to the intrusion of warm seawater to the fjord, with the values of temperature higher than the surface mean value (Fig. 3a). The subsurface warm water also upwelled up to surface.

Salinity values range between 34 and 35 ppt (Supplementary Fig. S1) with the current speed from 0.02 to 0.3 ms^−1^ and the flow direction is seen to be in the Southeast and Northwest which follow the topographic steering of the fjord (Supplementary Fig. S1). This unusual increase in water temperature and salinity in the entire water column was caused by the unidirectional (to the Southeast) flow of warm seawater into the Kongsfjorden, and led to the sea ice melting, the sound of which has been recorded on 18th December 2015.

Figure 3b presents time series of water temperature anomalies in the Kongsfjorden during winter 2016–17. Throughout the time series, a strong positive water temperature anomaly is observed in December. The average positive surface water temperature anomaly of 1.5 °C is higher in winter 2016–17 as compared to that of 2015–16. This stepwise increase of water temperature along with the salinity above 34.9 ppt indicate a persistent warm seawater flow (Supplementary Fig. S2) towards the Kongsfjorden associated with prior to and during the passage of extreme cyclonic storms. The current direction is typically in Southeast and Northwest which follows the neritic incursion and excursion of water at the Kongsforjden. Water temperature gradually increases from 1st December 2016 with a maximum positive anomaly of 3 °C by 9th and 13th December at the depth of 30 m. This rapid rise of temperature in the Kongsfjorden through the process of horizontal advection, influenced the entire water column of the fjord, and resulted in sea ice melting as seen in the noise recorded (Fig. 1b).

The variability of salinity along with current speed and direction are shown in Supplementary Figs. S3 and S4 during winter 2017–18 and winter 2018–19, respectively. It is observed that the value of salinity increases due to the intrusion of seawater in the fjord (Supplementary Fig. S3). The positive temperature anomaly is observed from 1st December to 20th December 2017 (Fig. 3c), associated with the prior period of passage of extreme cyclone event, and sea ice melting noise have been recorded. However, no strong positive temperature anomaly (Fig. 3d) and no sea ice melting sounds are observed in winter 2018–19 (Fig. 1d).

## Discussion

The main finding of this study is the sea ice melting sound in winter and their interannual variability in the Kongsfjorden Arctic. The sounds produced by the sea ice melting with spectral peak in the range of 1–3 kHz is similar to the sounds recorded in the summer glacierized fjord at Icy Bay, Alaska and laboratory experiments^31–33^. However, the spectrogram in Fig. 1 shows that the spectral content extends in the frequency range up to 10 kHz. Our study shows that the maximum spectral content lies between 1 and 3 kHz, and the noise spectrum level extending to higher frequencies, are mainly due to the breaking of multiple air bubbles in varying sizes during the sea ice melting. It has been reported that the size and content of air bubbles vary during the period of ice growth^40^. The content of entrapped air bubbles in sea ice is different from the glacier ice due to variations in depth and internal pore pressure^29,41^. It has been studied that low internal pore pressure generates smaller air bubbles, and high internal pore pressure makes it larger air bubbles^29,41^.The sound radiated by larger air bubble peaks at lower frequency and the sound from smaller air bubbles extends to higher frequency^42^. It was also experimented that acoustic signals emitted by multiple bubble release events range over a frequency band 6–12 kHz^33^.

This study reveals that sea ice melting sounds are anomalous in winter and related to the high values of surface air temperature as well as the strong positive anomaly of water temperature. On 18th December, 2015 the recordings of sea ice melting sounds are due to the intrusion of warm seawater associated with the extreme cyclone event. However, on December 30th, 2015 the recordings of sea ice melting sounds are primarily caused by warming of surface air temperature (7 °C) which is associated with the passage of extreme North Atlantic windstorm event 'Frank'. A previous study showed that reduction in sea surface ice concentration on Svalbard is due to the warming surface air temperature associated with the extreme warming event^16^. The recent study also reveals that a high number of sea ice melting sounds are recorded during winter 2016–17, and they coincide with the positive anomaly of water temperature due to warm seawater entering the Kongsfjorden. A previous study^43^ attributed that the rapid decline of sea ice is due to the intense sea surface temperature anomalies associated with passage of extreme cyclone events during winter 2016. Here, we are able to clearly show such events in the Arctic, using in-situ acoustic measurements.

In general, sea ice melting sounds are recorded in summer, which depend on surface area of ice exposed to the water, air temperature, winds and water temperature^33^. The present study describes the observation of sea ice melting sound in winter, and the causative processes such as the high values of surface air temperature and intrusion of seawater associated with the passage of cyclones either prior to or during the extreme events. Winter sea ice melting sounds are significant in describing the reduction of young-sea ice as well as the first-year ice. This ceasing of sea ice growth during winter, increases the ocean ambient temperature, which can lead to greater warming over time and making the Arctic glacier fjords more sensitive. The decline of sea ice concentration in winter can disrupt normal ocean circulation as well as the global conveyor belt, and therefore leading to changes in global climate. This study also illustrates that in-situ passive acoustic monitoring for long term is an effective way to understand the rate of sea ice melting which has consequence in the radiation budget and also Arctic marine ecosystems.

## Materials and methods

**Figure 4:**
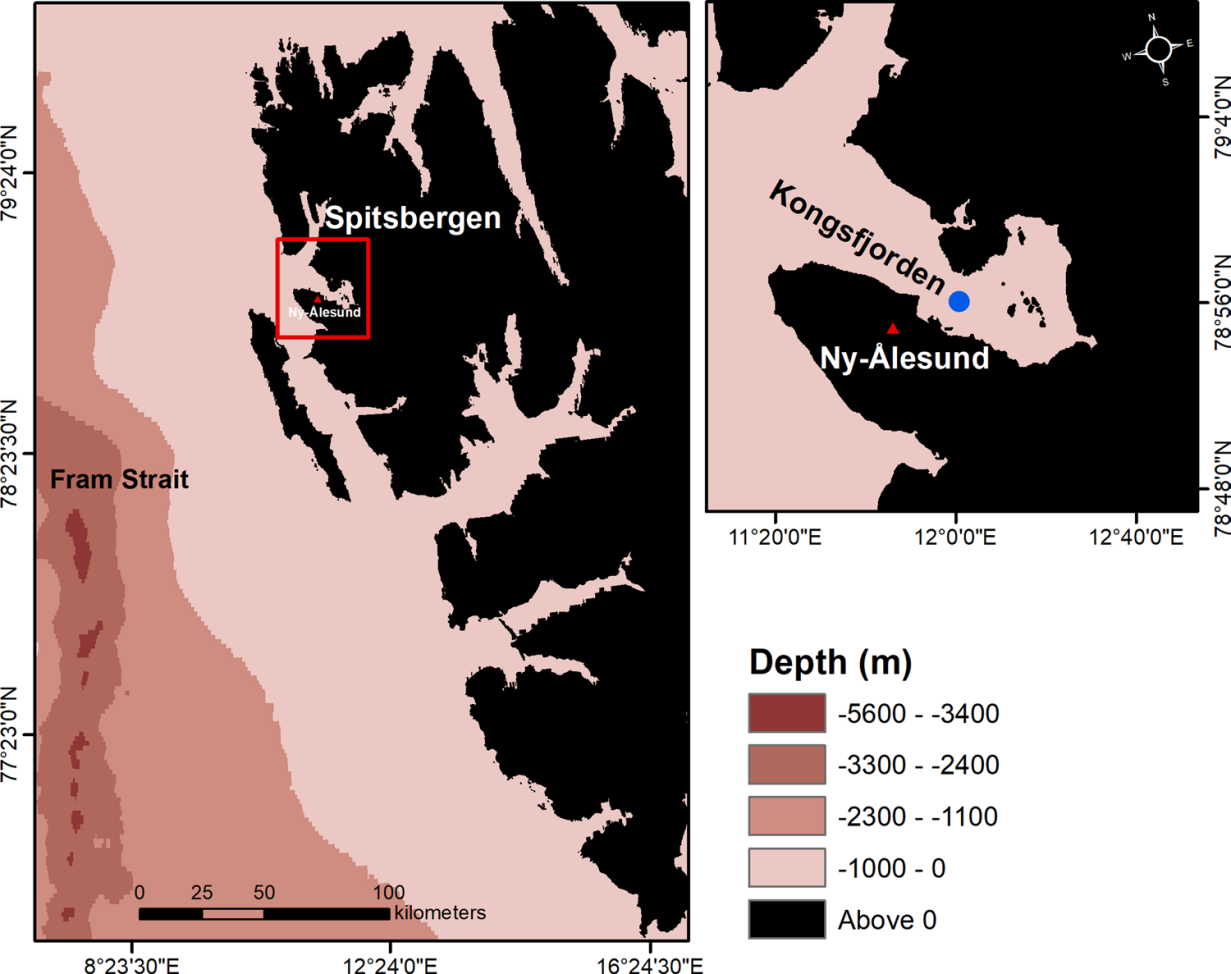
Deployment location of IndArc mooring at the Kongsfjorden is indicated in blue filled dot. This figure is generated using ArcMap 10.8.1 (https://desktop.arcgis.com/en/arcmap/latest/get-started/introduction/whats-new-in-arcgis.htm).

The underwater ambient noise measurement system connected with IndArc mooring^39^ was deployed in the Kongsfjorden (Fig. 4), Arctic Ocean, at an ocean depth of 198 m in July 2015. It is maintained since then by retrieval and redeployment in July every year, and thus four years of data have been obtained so far. The system consists of Cetacean make omni-directional hydrophone with a bandwidth of 15 Hz–40 kHz (https://www.cetaceanresearch.com/index.html), data acquisition and battery power packs which was positioned at a depth of 40 m.

The noise data was acquired at a sampling rate of 50 kHz for a duration of 60 s once in every 3 h for the period 2015–16. From July 2016 onwards, the data has been acquired once in every hour, at a sampling rate of 25 kHz for a duration of 180 s. The preamplifier gain of hydrophone is 20 dB with a receiving sensitivity of − 185 dB re 1 V/µPa. The ambient noise was estimated by using Welch’s power spectral density (https://www.mathworks.com/help/signal/ref/pwelch.html). The data is segmented into smaller portions to obtain multiple spectra using a Hamming window and 2048-point FFT with 50% overlap yielding 24.4 Hz bins. In this study, the focus is on analysis of noise during midwinter period in the four-year measurements i.e. December 2015 to January 2019. In order to understand the water temperature anomalies in the Kongsfjorden, the hourly time series data of 7 CT sensors (SBE 37-IMP MicroCAT, https://www.seabird.com) positioned in the mooring system over the range 30–170 m during winter 2015–16 and 2016–17, and a range of 60–170 m during winter 2017–18 and 2018–19 have been considered. The current speed and direction were recorded in every 20 min using upward facing Acoustic Doppler Current Profiler (ADCP, Teledyne RDI 150 kHz, https://www.teledynemarine.com/rdi/), located at 170 m depth during winter 2015–16, and at 76 m during winter 2016–17, 2017–18 and 2018–19 with a vertical resolution of 4 m at IndArc mooring. The hourly meteorological parameters, particularly surface pressure and surface air temperature were used from the Ny-Alesund weather station (https://www.yr.no/place/Norway/Svalbard/Ny%C3%85lesund/detailed_statistics.html), which is closest (∼ 2.5 km) to the IndArc mooring station (Fig. 4). These atmospheric parameters are used to represent the number of occurrences of extreme low pressure events as well as the fluctuation of surface air temperature at the mooring location. In this study, we have considered the occurrences of extreme low pressure event below a threshold of 985 hPa.

## Supplementary information

Supplementary Information 1.

Supplementary Information 2.
