# Supplementary material for: Underwater sound to probe sea ice melting in the Arctic during winter
Source: Sci Rep. 2020 Sep 29;10:16047. doi: 10.1038/s41598-020-72917-4 (PMC7524793; doi:10.1038/s41598-020-72917-4)
Supplement: Supplementary file 2 — Supplementary Information 2. [file 41598_2020_72917_MOESM2_ESM.docx]

Supplementary Materials for

**Underwater sound to probe sea ice melting in the Arctic during winter**

Madan M. Mahanty^1*^, Latha G^1^, Venkatesan R^1^, Ravichandran M^2^, Atmanand M. A^1^, Thirunavukarasu A^1^ and Raguraman G^1^

*Correspondence to: [mmmahanty@gmail.com](mailto:mmmahanty@gmail.com)

**This word file includes:**

Figs. S1 to S4

Caption for Audio S1


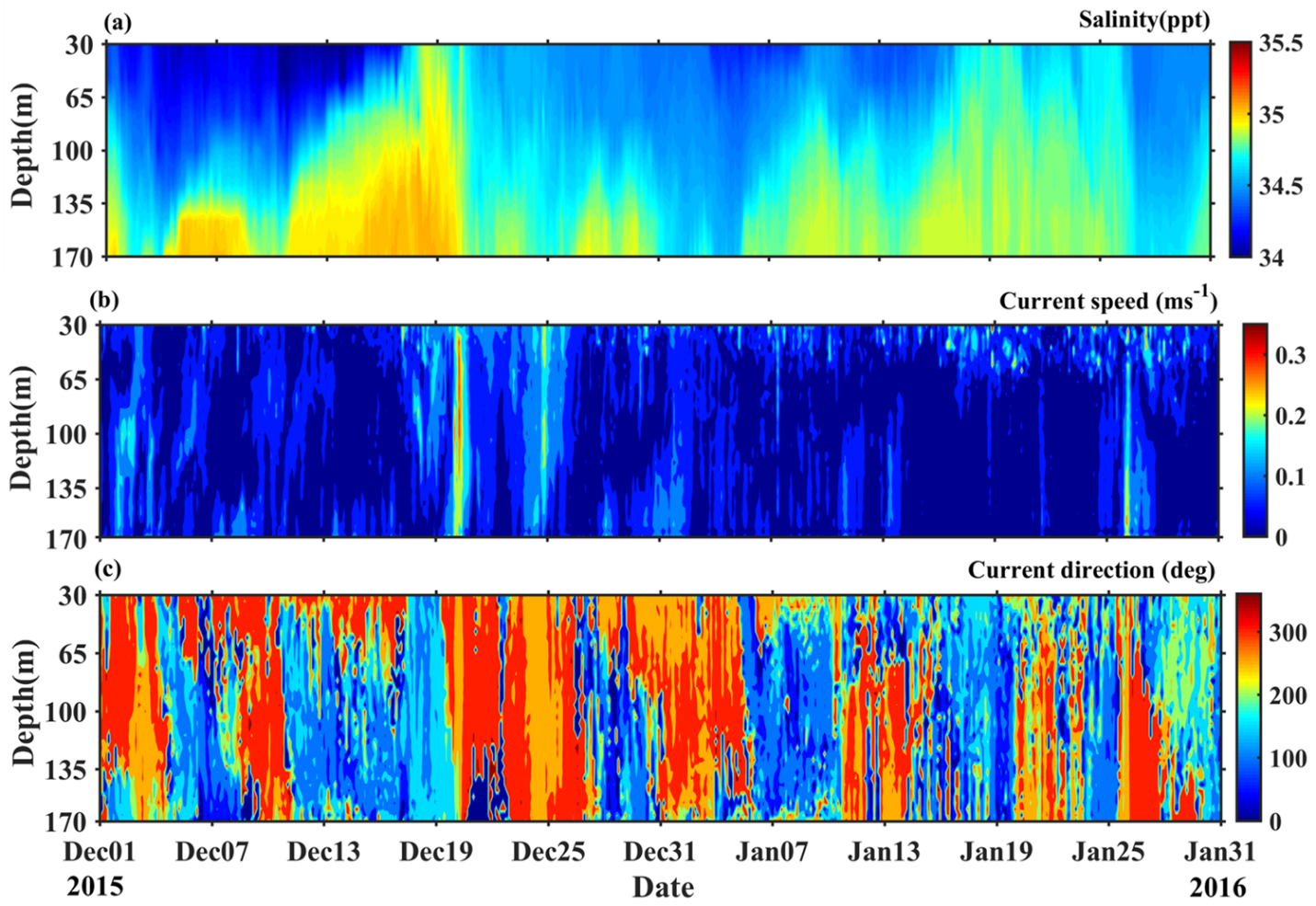


**Fig. S1.** *In-situ* oceanographic data for (a) salinity (ppt), (b) current speed (ms^-1^), and (c) current direction (deg) in the Kongsfjorden during winter 2015-16.


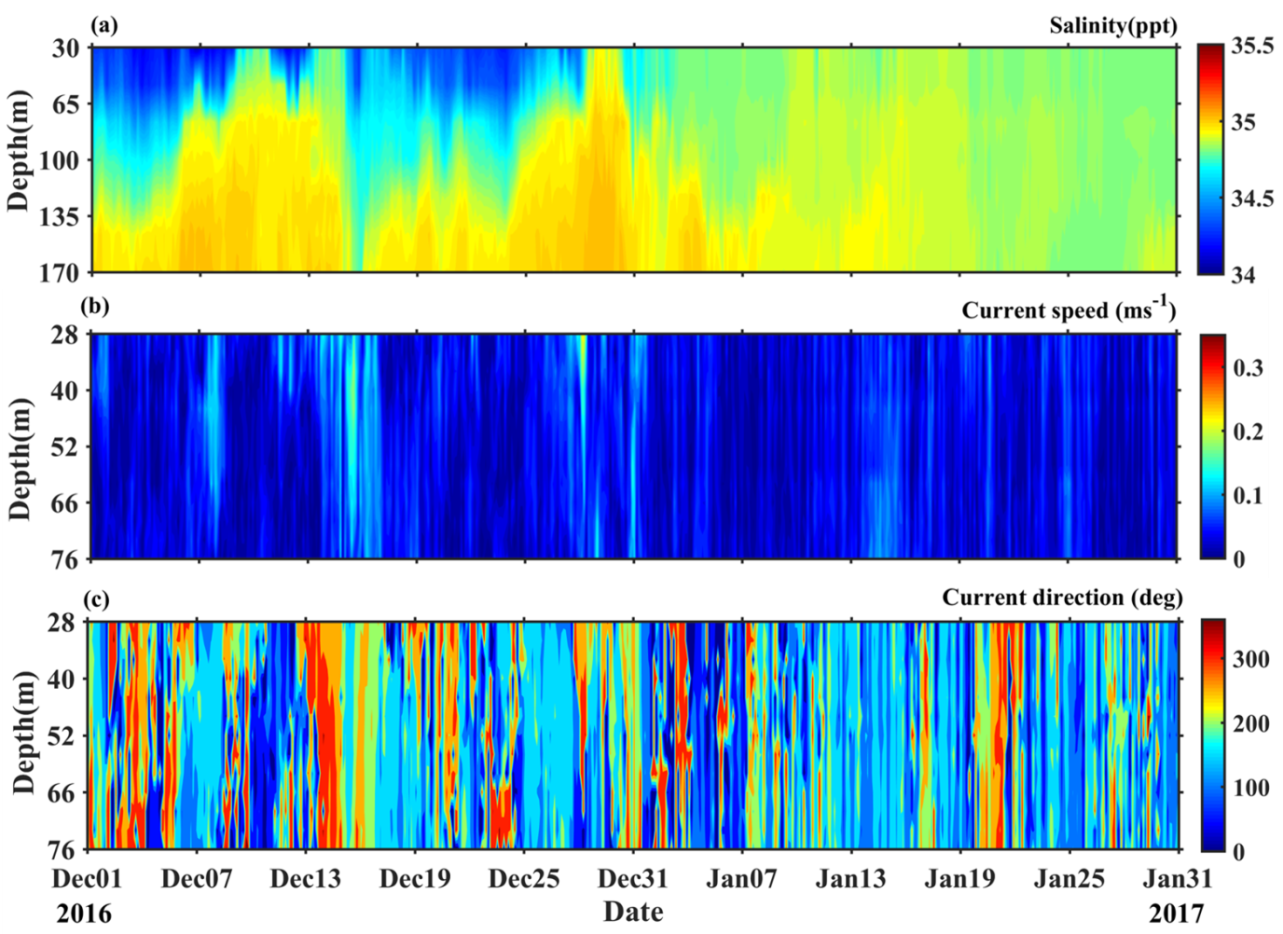


**Fig. S2.** *In-situ* oceanographic data for (a) salinity (ppt), (b) current speed (ms^-1^), and (c) current direction (deg) in the Kongsfjorden during winter 2016-17.


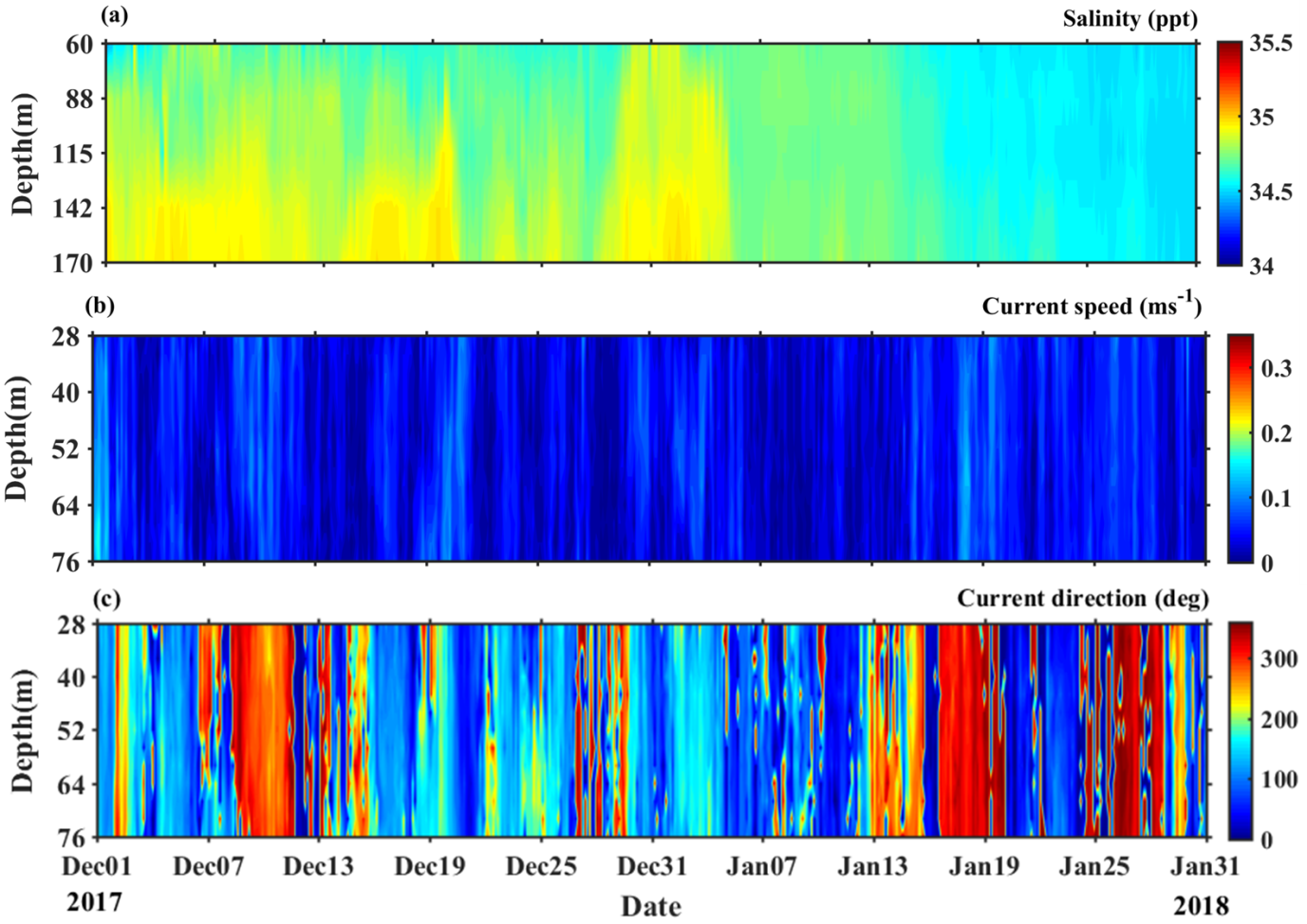


**Fig. S3**. *In-situ* oceanographic data for (a) salinity (ppt), (b) current speed (ms^-1^), and (c) current direction (deg) in the Kongsfjorden during winter 2017-18.


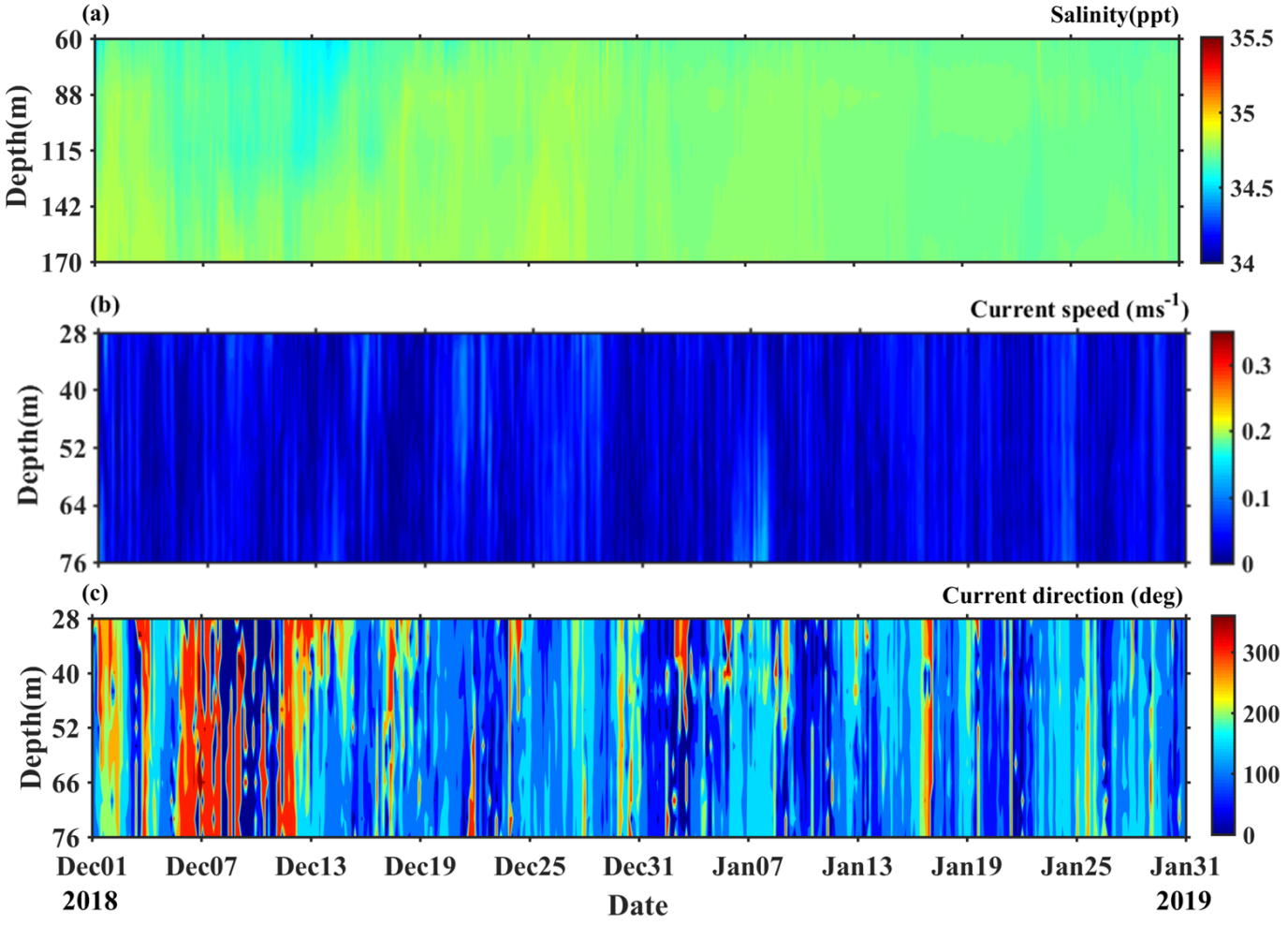


**Fig. S4.** *In-situ* oceanographic data for (a) salinity (ppt), (b) current speed (ms^-1^), and (c) current direction (deg) in the Kongsfjorden during winter 2018-19.

**Audio-S1.** Recording of underwater sound produced by the sea ice melting in Kongsfjorden during winter.
